# Advances in the Diagnosis and Disease-Modifying Management of Transthyretin Amyloid Cardiomyopathy: A Narrative Review

**DOI:** 10.7759/cureus.103843

**Published:** 2026-02-18

**Authors:** Vasudeva Vijaykumar

**Affiliations:** 1 Internal Medicine, Independent Research, Mysore, IND

**Keywords:** attr cardiomyopathy, cardiac amyloidosis, disease-modifying therapy, multimodal cardiac imaging, non-invasive diagnosis, radionuclide scintigraphy, rna-silencing therapies, transthyretin amyloid cardiomyopathy, transthyretin stabilizers

## Abstract

Transthyretin amyloid cardiomyopathy (ATTR-CM) is an increasingly recognized cause of heart failure that has historically been underdiagnosed due to nonspecific clinical presentation and limited awareness. Advances in non-invasive diagnostic modalities have transformed the identification of ATTR-CM, allowing accurate diagnosis without routine reliance on endomyocardial biopsy and enabling earlier-stage disease detection. These developments have coincided with a rapidly evolving therapeutic landscape, marked by the introduction of disease-modifying treatments that directly target transthyretin stability or production rather than providing solely supportive care. Meaningful improvements in clinical outcomes with transthyretin stabilizers and gene-silencing therapies have been seen, fundamentally altering disease management and prognosis. Despite this progress, challenges persist in optimizing screening strategies, ensuring timely diagnosis, and integrating emerging therapies into routine clinical practice. This narrative review synthesizes contemporary evidence on the pathobiology, diagnosis, and treatment of ATTR-CM, highlighting recent advances and persistent gaps in care. While it provides a qualitative overview of the current landscape to improve patient outcomes, it does not constitute a formal systematic review or meta-analysis.

## Introduction and background

Transthyretin amyloid cardiomyopathy (ATTR-CM) is an infiltrative myocardial disease resulting from extracellular deposition of misfolded transthyretin amyloid fibrils within the cardiac interstitium, leading to progressive ventricular dysfunction and heart failure [1,2]. Transthyretin is a transport protein synthesized primarily in the liver, and its instability, either due to pathogenic gene variants or age-related conformational changes, underlies amyloid fibril formation and tissue deposition [2,3]. ATTR-CM occurs in both hereditary and wild-type forms, which differ in age of onset and systemic involvement but share a cardiac phenotype characterized by restrictive physiology and progressive clinical deterioration [3,4].

Clinically, ATTR-CM is increasingly recognized as an important cause of heart failure in older adults. Evidence from observational cohorts and contemporary reviews indicates that wild-type ATTR-CM is prevalent among elderly patients with heart failure, particularly those with preserved or mildly reduced ejection fraction and unexplained left ventricular hypertrophy [1,5]. Despite this, ATTR-CM has historically been underdiagnosed, largely due to overlap with more common cardiovascular conditions such as hypertensive heart disease, hypertrophic cardiomyopathy, and age-related diastolic dysfunction [5,6]. As a result, many patients have traditionally been diagnosed at advanced stages, when therapeutic options were limited and outcomes poor [7].

The pathophysiological consequences of transthyretin amyloid deposition include increased myocardial stiffness, impaired ventricular relaxation, atrial dysfunction, and involvement of the cardiac conduction system [1,2]. These changes manifest clinically as progressive exertional intolerance, heart failure symptoms, arrhythmias, and conduction disturbances. Importantly, ATTR amyloidosis is increasingly recognized as a multisystem disorder, with extracardiac manifestations that may precede cardiac involvement and support earlier clinical suspicion [4,8].

Over the past decade, the diagnostic approach to ATTR-CM has evolved substantially. Advances in non-invasive imaging have enabled accurate identification of ATTR-CM without routine reliance on endomyocardial biopsy in appropriately selected patients [9-11]. Echocardiography and cardiac magnetic resonance imaging (MRI) provide structural and functional clues suggestive of infiltrative cardiomyopathy. At the same time, bone-avid radionuclide scintigraphy has emerged as a pivotal tool for confirming transthyretin amyloid deposition when light-chain amyloidosis is excluded [9,10,12]. These developments have facilitated broader screening initiatives and improved diagnostic confidence in routine clinical practice [5,6].

In parallel with diagnostic advances, the therapeutic landscape of ATTR-CM has undergone a fundamental transformation. Disease-modifying therapies targeting transthyretin stability or production have demonstrated the ability to slow disease progression and improve clinical outcomes, representing a shift from supportive heart failure management to mechanism-based treatment [13-16]. Contemporary reviews emphasize that earlier diagnosis is critical to maximizing therapeutic benefit, as treatment efficacy appears greatest when initiated before advanced myocardial dysfunction has developed [17,18].

Together, these diagnostic and therapeutic advances have repositioned ATTR-CM as a treatable cardiomyopathy rather than an irreversible cause of heart failure. However, challenges remain in achieving timely diagnosis, optimizing treatment selection, and integrating emerging therapies into everyday clinical practice [7,19]. This narrative review synthesizes current evidence on the pathophysiology, diagnosis, and management of ATTR-CM, with a focus on recent advances and ongoing gaps in care.

Literature search strategy

A targeted literature search was conducted to identify relevant studies addressing the diagnosis and disease-modifying management of transthyretin amyloid cardiomyopathy. The PubMed database was searched for articles published in English using combinations of keywords and Medical Subject Headings (MeSH), including “transthyretin amyloid cardiomyopathy,” “ATTR cardiomyopathy,” “cardiac amyloidosis,” “diagnosis,” “radionuclide scintigraphy,” “tafamidis,” “transthyretin stabilizers,” and “RNA-silencing therapies.” Emphasis was placed on recent publications and studies relevant to contemporary clinical practice.

The search focused on review articles, randomized clinical trials, post hoc analyses, and real-world observational studies that addressed diagnostic strategies, multimodal imaging, and disease-modifying therapies in transthyretin amyloid cardiomyopathy. Reference lists of selected articles were manually screened to identify additional relevant publications. Articles were selected based on relevance to current diagnostic pathways and therapeutic management, with emphasis on studies informing clinical decision-making in the modern treatment era. Given the narrative nature of this review, predefined inclusion and exclusion criteria, structured study selection counts, and formal risk-of-bias assessment were not applied, and no quantitative synthesis was performed.

## Review

Overview: pathophysiology, clinical features, and epidemiology

ATTR-CM develops as a consequence of progressive myocardial infiltration by transthyretin amyloid fibrils, resulting in structural remodeling and functional impairment of the heart [1,2]. The pathogenic process is initiated by destabilization of the transthyretin tetramer, leading to dissociation into monomers that misfold and aggregate into insoluble amyloid fibrils [2,3]. These fibrils accumulate within the myocardial extracellular matrix, causing increased wall thickness, reduced ventricular compliance, and impaired diastolic relaxation, collectively defining the restrictive phenotype characteristic of ATTR-CM [1,7].

As amyloid deposition progresses, involvement extends beyond the ventricular myocardium to the atria and cardiac conduction system. Atrial infiltration contributes to impaired atrial mechanical function and predisposes to atrial arrhythmias, whereas conduction system involvement results in atrioventricular block and other conduction disturbances, which are frequently observed in patients with ATTR-CM [2,7]. These pathological changes lead to a gradual decline in cardiac reserve, consistent with the insidious onset and progressive nature of clinical symptoms [3].

Clinically, ATTR-CM presents with a spectrum of cardiac manifestations that evolve over time. Early disease is often characterized by exertional dyspnea and reduced exercise tolerance, reflecting impaired diastolic filling and elevated filling pressures [1]. As the disease advances, patients develop overt heart failure symptoms, recurrent hospitalizations, and progressive functional limitation, with systolic dysfunction occurring in more advanced stages [7,19]. Arrhythmias and conduction abnormalities further complicate the clinical course and contribute to morbidity [2].

Importantly, contemporary evidence increasingly frames transthyretin amyloid cardiomyopathy as a heart failure syndrome rather than a rare cardiomyopathy subtype. ATTR-CM may present across the spectrum of heart failure phenotypes, including both preserved and reduced ejection fraction, reflecting progressive myocardial infiltration and remodeling. Recognition of this phenotypic heterogeneity has important implications for clinical suspicion and diagnostic evaluation, particularly in older patients presenting with unexplained heart failure symptoms [20].

ATTR-CM is increasingly recognized as a systemic disorder rather than an isolated cardiac condition. Extracardiac manifestations are common and may precede the onset of cardiac symptoms by several years. Musculoskeletal features such as bilateral carpal tunnel syndrome and lumbar spinal stenosis, along with peripheral and autonomic neuropathy, have been frequently described in both hereditary and wild-type disease [3,4]. Ocular involvement, including vitreous opacities and other visual disturbances, has also been reported and may serve as an additional diagnostic clue in patients with multisystem involvement [8]. Recognition of these extracardiac features is clinically important, as they may raise suspicion for underlying transthyretin amyloidosis.

From an epidemiological perspective, ATTR-CM has transitioned from a perceived rare disease to a condition increasingly identified in routine cardiology practice. Wild-type ATTR-CM predominantly affects older adults and has been reported in substantial proportions of elderly patients with heart failure, particularly those with preserved or mildly reduced ejection fraction and unexplained ventricular hypertrophy [1,5]. Hereditary ATTR-CM exhibits geographic and ethnic variability, reflecting the distribution of pathogenic transthyretin mutations, but may also present later in life with a predominantly cardiac phenotype [3].

Despite growing recognition, ATTR-CM remains underdiagnosed, and the true disease prevalence is likely underestimated. Contemporary reviews emphasize that delayed diagnosis contributes to advanced disease at presentation and may limit the potential benefits of disease-modifying therapy [6,7]. Improved understanding of disease pathophysiology, coupled with awareness of characteristic clinical and epidemiological features, is therefore essential to facilitate earlier identification and intervention.

Diagnostic approach and screening

The diagnosis of ATTR-CM has undergone a major transformation with the development of structured, non-invasive diagnostic pathways that integrate clinical evaluation, laboratory testing, and multimodal cardiac imaging [1,6]. Historically, ATTR-CM was frequently diagnosed late in the disease course, often requiring endomyocardial biopsy for confirmation. Contemporary diagnostic strategies now prioritize early recognition and non-invasive confirmation, enabling earlier identification of affected patients [9,10].

Clinical Suspicion and Identification of Red Flags

Clinical suspicion remains the critical first step in the diagnostic process. ATTR-CM should be considered in patients presenting with heart failure symptoms in the setting of unexplained left ventricular hypertrophy, particularly when conventional etiologies such as hypertension or valvular disease do not fully account for the degree of myocardial thickening [5,6]. Reviews emphasize that ATTR-CM is frequently encountered in older adults with heart failure with preserved or mildly reduced ejection fraction, a population in which diagnostic vigilance is especially important [1].

Extracardiac manifestations play an important role in raising diagnostic suspicion. Musculoskeletal features such as bilateral carpal tunnel syndrome and lumbar spinal stenosis, as well as peripheral and autonomic neuropathy, are commonly reported and may precede the onset of cardiac symptoms by several years [3,4]. Ocular involvement, including vitreous opacities and other visual disturbances, has also been described and may provide an additional clue to underlying transthyretin amyloidosis in selected patients [8]. Recognition of these systemic features is emphasized across contemporary reviews as an important component of clinical suspicion [7].

Biomarkers may provide additional supportive information during the diagnostic evaluation of suspected ATTR-CM. Chronic elevation of cardiac troponin and disproportionate increases in natriuretic peptides reflect ongoing myocardial injury and wall stress and may strengthen clinical suspicion when interpreted in conjunction with imaging findings. However, biomarker levels may be influenced by renal dysfunction and comorbid conditions, underscoring their role as adjunctive rather than definitive diagnostic tools [20].

Role of Echocardiography in Initial Evaluation

Echocardiography is typically the first-line imaging modality for patients being evaluated for suspected ATTR-CM. Characteristic findings include increased ventricular wall thickness, biatrial enlargement, restrictive filling patterns, and reduced longitudinal myocardial deformation consistent with an infiltrative cardiomyopathy [1,12]. Although these findings are not specific to amyloidosis, their presence in appropriate clinical contexts often prompts further diagnostic evaluation [6].

Advanced echocardiographic techniques, including strain imaging, may demonstrate patterns suggestive of amyloid involvement and may provide additional supportive information [12]. However, reviews emphasize that echocardiography alone is insufficient for definitive diagnosis and must be interpreted in conjunction with other imaging modalities and laboratory testing [1].

Cardiac MRI

Cardiac MRI plays a complementary role in the diagnostic evaluation of ATTR-CM by enabling detailed tissue characterization and assessment of myocardial involvement. Typical features include diffuse late gadolinium enhancement, abnormal myocardial nulling patterns, and expansion of the extracellular volume fraction, reflecting diffuse amyloid infiltration of the myocardium [11,12]. These findings are characteristic of cardiac amyloidosis and may indicate myocardial involvement even in early disease stages.

Despite its diagnostic utility, cardiac MRI cannot reliably distinguish transthyretin amyloid from light-chain amyloid cardiomyopathy. Accordingly, contemporary diagnostic algorithms emphasize the need to integrate cardiac MRI findings with laboratory testing and radionuclide imaging to establish amyloid type [6,11].

Radionuclide Scintigraphy and Non-Invasive Confirmation

Bone-avid radionuclide scintigraphy using technetium-99m-labeled tracers has emerged as a cornerstone of non-invasive diagnosis for ATTR-CM. When myocardial tracer uptake is present and monoclonal protein disorders are excluded, radionuclide scintigraphy allows for confident diagnosis of transthyretin amyloid cardiomyopathy without the need for endomyocardial biopsy [9,10].

Reviews emphasize the importance of standardized acquisition protocols and careful interpretation to ensure diagnostic accuracy and avoid misclassification, particularly in patients with concomitant plasma cell dyscrasias [9,10]. Specifically, diagnostic algorithms now incorporate renal-adjusted thresholds for serum free light chain ratios (e.g., up to 3.1 in advanced renal failure) to prevent false-positive diagnoses of light-chain amyloidosis in older patients with comorbid kidney disease [6,10]. The widespread adoption of radionuclide imaging has reduced reliance on invasive diagnostic procedures and supported broader adoption of non-invasive diagnostic pathways [6].

Screening Strategies and High-Risk Populations

Growing recognition of ATTR-CM has led to increasing interest in targeted screening strategies. Reviews highlight the potential value of screening high-risk populations, including older adults with heart failure, patients with unexplained ventricular hypertrophy, and individuals with aortic stenosis or systemic features suggestive of amyloidosis [5,6]. Such approaches aim to identify disease earlier, before advanced myocardial dysfunction limits therapeutic benefit.

Despite these advances, contemporary analyses note variability in screening practices and the absence of universally accepted screening recommendations [1,7]. Ongoing efforts to refine screening criteria and integrate systematic diagnostic approaches into routine clinical practice are viewed as essential to address persistent underdiagnosis [5].

Integration of Diagnostic Modalities

Modern diagnostic algorithms for ATTR-CM emphasize integration of clinical assessment, laboratory testing, and multimodal imaging rather than reliance on a single diagnostic test [1,6]. This integrated approach allows clinicians to establish a diagnosis with greater confidence, stratify disease severity, and inform clinical decision-making and subsequent management.

Overall, advances in diagnostic imaging and structured screening strategies have fundamentally reshaped the evaluation of ATTR-CM. Continued efforts to improve awareness, standardize diagnostic pathways, and expand access to non-invasive testing remain critical to optimizing patient outcomes [7,19]. The key non-invasive diagnostic modalities used in the evaluation of transthyretin amyloid cardiomyopathy, along with their roles and limitations, are summarized in Table 1.

**Table 1:** Non-invasive diagnostic tools in transthyretin amyloid cardiomyopathy ATTR-CM: transthyretin amyloid cardiomyopathy; AL: light-chain amyloidosis.; 99mTc: technetium-99m

Diagnostic Modality	Key Findings in ATTR-CM	Diagnostic Role	Key Limitations	Source Basis
Transthoracic echocardiography	Increased ventricular wall thickness, biatrial enlargement, restrictive filling pattern, abnormal myocardial deformation	First-line screening and suspicion generation	Findings are not specific to amyloidosis	Tschöpe et al. [1]; Kancerek et al. [12]
Speckle-tracking echocardiography	Reduced longitudinal strain with characteristic deformation patterns	Supports the suspicion of infiltrative cardiomyopathy suspicion	Cannot confirm amyloid type	Kancerek et al. [12]
Cardiac magnetic resonance imaging	Diffuse late gadolinium enhancement, abnormal myocardial nulling, increased extracellular volume.	Sensitive detection of cardiac amyloidosis	Cannot distinguish ATTR from AL amyloidosis	Marchionni et al. [11]; Kancerek et al. [12]
Radionuclide bone scintigraphy (99mTc tracers)	Myocardial tracer uptake in ATTR-CM	Non-invasive confirmation when monoclonal protein is excluded	Requires strict protocol adherence	Hanna et al. [9]; Benz et al. [10]
Laboratory evaluation (serum/urine studies)	Exclusion of monoclonal gammopathy	Essential to differentiate ATTR from AL amyloidosis	Does not confirm ATTR independently	Hanna et al. [9]; Benz et al. [10]

Disease-modifying therapies

The therapeutic approach to ATTR-CM has evolved markedly with the advent of agents that directly target the molecular mechanisms underlying amyloid formation and deposition [3,17]. Historically, management was limited to conventional heart failure therapies, which provided symptomatic relief but did not alter disease progression. Advances in understanding transthyretin biology have led to the development of disease-modifying therapies that either stabilize the transthyretin tetramer or reduce hepatic transthyretin production, thereby addressing the upstream drivers of amyloid cardiomyopathy [18,21]. Contemporary reviews emphasize that the timely initiation of these therapies is critical, as irreversible myocardial damage may limit their effectiveness in advanced stages of disease [7,17]. Importantly, contemporary management frameworks emphasize that disease-modifying therapies should be integrated within comprehensive heart failure care rather than used in isolation, underscoring the importance of early diagnosis and individualized treatment strategies [20].

Transthyretin Stabilization Therapy: Tafamidis

Transthyretin stabilizers act by binding to the thyroxine-binding sites of the transthyretin tetramer, increasing kinetic stability and preventing dissociation into amyloidogenic monomers [3,18]. Tafamidis was the first agent in this class to demonstrate clinical efficacy in ATTR-CM. Clinical trial data showed that tafamidis therapy was associated with improved clinical outcomes, including reductions in mortality and cardiovascular-related hospitalizations, establishing its role as a foundational disease-modifying therapy [13].

Beyond primary trial outcomes, post hoc analyses showed that tafamidis was associated with a reduction in functional decline and preserved cardiac structure, supporting a disease-modifying role beyond symptomatic management [13]. These findings align with mechanistic expectations of tetramer stabilization and underscore the importance of intervening early in the disease course.

Real-world studies have provided complementary evidence supporting the effectiveness of tafamidis in routine clinical practice. Observational cohorts have reported clinical benefit across broader patient populations, including older adults and patients with more advanced functional impairment, although outcomes appear to be influenced by baseline disease severity and timing of therapy initiation [14]. These observations reinforce the clinical importance of early diagnosis and prompt initiation of transthyretin-stabilizing therapy [17,18].

Next-Generation Transthyretin Stabilization With Acoramidis

Acoramidis is a next-generation transthyretin stabilizer developed to achieve near-complete kinetic stabilization of the transthyretin tetramer, addressing limitations observed with earlier agents [3]. By binding with high affinity to transthyretin, acoramidis enhances tetramer stability and reduces dissociation into amyloidogenic monomers, thereby targeting a critical step in amyloid fibril formation [17,18].

Clinical evidence supporting acoramidis has emerged from the ATTRibute-CM trial program [15]. Long-term data from the open-label extension demonstrated sustained efficacy and a favorable safety profile with continued treatment, suggesting durable disease stabilization over extended follow-up periods. Furthermore, treatment with acoramidis elicits a prompt and sustained increase in serum transthyretin levels, which serves as an accessible in vivo biomarker of tetramer stabilization and has been associated with improved survival [15]. These findings are particularly relevant given the chronic and progressive nature of ATTR-CM, where long-term therapeutic durability is a key clinical consideration.

Contemporary reviews highlight acoramidis as an important addition to the transthyretin stabilizer class, with potential applicability across a range of disease stages [17,18]. Its role may be especially relevant in patients with early to intermediate disease, where maximal tetramer stabilization could translate into meaningful clinical benefit, although optimal patient selection continues to be refined [3,17].

RNA-Silencing Therapies Targeting Transthyretin Production

RNA-silencing therapies represent a mechanistically distinct approach to disease modification by directly suppressing hepatic transthyretin synthesis [21]. These agents reduce circulating levels of amyloidogenic transthyretin protein, thereby limiting ongoing amyloid deposition and potentially altering disease progression at its source.

Vutrisiran, a subcutaneously administered small interfering RNA, has demonstrated clinically relevant effects in patients with transthyretin amyloidosis with cardiomyopathy. Clinical trial analyses reported favorable effects on cardiac structure and function, reflecting attenuation of myocardial remodeling and disease progression [16]. These findings provide direct clinical evidence that transthyretin-lowering strategies can influence cardiac manifestations of amyloid disease.

Contextual analyses of recent clinical trial data emphasize that RNA-silencing therapies may be particularly beneficial in patients with established amyloid burden or ongoing transthyretin production despite tetramer stabilization [22]. However, contemporary reviews also highlight unresolved questions about long-term outcomes, durability of response, and optimal integration with transthyretin stabilizers, underscoring the need for further comparative and longitudinal studies [17,18].

Clinical Integration and Real-World Considerations

Integrating disease-modifying therapies into routine clinical practice requires consideration of disease stage, comorbid conditions, and patient-specific factors [3,18]. Management of these comorbidities requires specific vigilance; for instance, anticoagulation is recommended for atrial fibrillation regardless of CHA2DS2-VASc score because of the high thrombotic risk, whereas conventional heart failure therapies, such as beta-blockers, are often poorly tolerated due to restrictive physiology [17,19,20]. Real-world analyses have identified barriers to optimal therapy utilization, including high treatment costs, limited access outside specialized centers, and diagnostic delays that restrict eligibility for disease-modifying interventions [23]. These challenges may disproportionately affect older patients and those with advanced disease, potentially limiting the population-level impact of available therapies [19,23]. Despite these costs, a recent meta-analysis highlighted the substantial value of these agents, estimating a number needed to treat (NNT) of only 10 to prevent one death over two years, underscoring their profound efficacy in this high-risk population [23].

Contemporary reviews emphasize the need for structured care pathways and multidisciplinary management to ensure timely diagnosis, appropriate therapy selection, and longitudinal follow-up [7,19]. As the therapeutic landscape continues to expand, comparative effectiveness studies and long-term outcome data will be essential for refining treatment algorithms and optimizing outcomes for patients with transthyretin amyloid cardiomyopathy. The currently available disease-modifying therapies for transthyretin amyloid cardiomyopathy, including their mechanisms of action, supporting clinical evidence, and key clinical considerations, are summarized in Table 2.

**Table 2:** Disease-modifying therapies for transthyretin amyloid cardiomyopathy ATTR-CM: transthyretin amyloid cardiomyopathy Therapy selection should consider overall disease stage, comorbid conditions, and treatment accessibility when interpreting the therapeutic options summarized in this table

Therapeutic Class	Agent	Mechanism of Action	Evidence Base	Clinical Considerations	Source Basis
Transthyretin stabilizer	Tafamidis	Stabilizes transthyretin tetramer, prevents monomer dissociation	Randomized trials and post hoc analyses	Benefit appears greatest when initiated early	Shah et al. [13]; Izumiya et al. [14]
Next-generation stabilizer	Acoramidis	Near-complete kinetic stabilization of transthyretin	Open-label extension of ATTRibute-CM	Long-term efficacy and safety have been demonstrated	Judge et al. [15]; Hellenbart et al. [17]
RNA-silencing therapy	Vutrisiran	Reduces hepatic production of transthyretin	Secondary analyses of clinical trials	Associated with favorable changes in cardiac structure and function	Jering et al. [16]; Girard & Sperry [22]
Emerging molecular therapies	RNA targeting / gene-editing strategies	Suppression of transthyretin synthesis	Early and translational-stage evidence	Long-term outcomes under investigation	Ioannou et al. [21]

Unmet needs and future directions

Despite substantial progress in the diagnosis and management of tATTR-CM, multiple unmet needs continue to limit optimal patient outcomes. Contemporary reviews emphasize that delayed diagnosis remains one of the most significant challenges in ATTR-CM, with many patients identified only after advanced myocardial involvement has occurred [5-7]. Diagnostic delays are frequently attributed to nonspecific clinical presentations, overlap with more prevalent cardiovascular conditions, and limited awareness of ATTR-CM outside specialized centers [1,5]. Addressing these barriers is critical, as disease-modifying therapies demonstrate greater benefit when initiated earlier in the disease course [17,18].

Although non-invasive diagnostic pathways have reduced reliance on endomyocardial biopsy, uncertainties remain regarding optimal screening strategies and patient selection. Reviews highlight variability in diagnostic algorithms across institutions and the absence of standardized screening recommendations for high-risk populations, such as older adults with heart failure or patients with coexisting conditions, including aortic stenosis [5,6,19]. Future efforts to refine screening criteria and integrate systematic diagnostic approaches into routine clinical practice may improve early identification and broaden access to disease-modifying therapies [1,19].

Therapeutic challenges also persist despite the availability of multiple disease-modifying agents. Contemporary analyses note that treatment selection is often influenced by disease stage, comorbidities, and logistical factors rather than direct comparative efficacy data [3,18]. The absence of head-to-head trials comparing transthyretin stabilizers with transthyretin-lowering therapies limits evidence-based decision-making and complicates individualized treatment strategies [17]. In addition, the long-term durability of therapeutic benefit and the optimal sequencing or combination of available agents remain areas of active investigation [22].

Real-world studies further underscore disparities in access to disease-modifying therapies. High treatment costs, reimbursement barriers, and limited availability outside tertiary referral centers have been identified as major obstacles to widespread implementation, potentially exacerbating inequities in care [19,23]. Importantly, available clinical trial and observational evidence suggest that patients with earlier-stage disease derive greater clinical benefit compared with those with advanced myocardial involvement, in whom irreversible structural remodeling may limit therapeutic response [13,17]. These findings underscore the importance of timely diagnosis, appropriate patient selection, and health system-level solutions, including streamlined referral pathways and policies that support equitable access to emerging therapies.

Recent state-of-the-art practice reviews further emphasize that addressing these gaps will require coordinated implementation strategies, including earlier recognition within general cardiology practice and integration of amyloid-specific therapies into established heart failure care pathways [20].

Future directions in ATTR-CM research include developing next-generation therapeutics to achieve more complete suppression of amyloid formation and enhanced clearance of existing amyloid deposits. Reviews discussing RNA-targeting and gene-editing strategies suggest that advances in molecular therapy may further expand the therapeutic landscape, although long-term safety and clinical efficacy require continued evaluation [21]. Additionally, improved understanding of disease heterogeneity and progression may facilitate more precise risk stratification and personalized treatment approaches [1,2].

Finally, contemporary reviews emphasize the importance of multidisciplinary care models and longitudinal disease monitoring to optimize outcomes in ATTR-CM [7,19]. Integration of cardiology, internal medicine, neurology, geriatrics, and advanced imaging expertise may enhance early recognition, diagnostic accuracy, and longitudinal management of patients with ATTR-CM. As awareness of ATTR-CM continues to grow, coordinated efforts across clinical, research, and policy domains will be essential to address remaining gaps and further improve patient care.

## Conclusions

ATTR-CM has undergone a fundamental transformation from a frequently overlooked cause of heart failure to a condition that can be accurately diagnosed and effectively treated when identified early. Advances in non-invasive diagnostic pathways have enabled reliable disease recognition in routine clinical practice, reducing reliance on invasive procedures and facilitating timely confirmation in appropriate patients. These developments have shifted the diagnostic paradigm toward earlier detection, which is increasingly critical in the era of disease-modifying therapy. The availability of therapies that directly target transthyretin stability and production has altered the natural history of ATTR-CM, moving management beyond symptomatic heart failure treatment toward mechanism-based intervention.

Evidence from clinical trials and real-world experience underscores the importance of initiating therapy before advanced myocardial dysfunction develops, reinforcing the close interdependence between early diagnosis and therapeutic benefit. As the therapeutic landscape continues to expand, careful integration of available agents into individualized care pathways remains essential. Despite these advances, challenges persist, including delayed diagnosis, variability in access to specialized care, and uncertainty regarding optimal treatment selection and sequencing. Addressing these gaps will require continued refinement of diagnostic strategies, broader awareness across clinical disciplines, and further research to guide long-term management. In this evolving treatment era, coordinated efforts to align early diagnosis with timely disease-modifying therapy offer the greatest opportunity to improve outcomes for patients with ATTR-CM.
